# Editorial: Mental fatigue and sport: from the lab to the field

**DOI:** 10.3389/fspor.2023.1213019

**Published:** 2023-05-09

**Authors:** Thiago Ribeiro Lopes, Leonardo de Sousa Fortes, Mitchell Robert Smith, Bart Roelands, Samuele Maria Marcora

**Affiliations:** ^1^ Laboratory of Exercise Physiology at Olympic Center of Training and Research, Department of Physiology, Federal University of São Paulo, São Paulo, Brazil; ^2^ São Paulo Association for Medicine Development, São Paulo, Brazil; ^3^ Graduate Program in Translational Medicine, Department of Medicine, Paulista School of Medicine, Federal University of São Paulo, São Paulo, Brazil; ^4^ Department of Physical Education, Federal University of Paraíba, João Pessoa, Brazil; ^5^ Discipline of Exercise and Sports Science, College of Engineering, Science and Environment, University of Newcastle, Ourimbah, NSW, Australia; ^6^ Active Living Research Program, Hunter Medical Research Institute, New Lambton Heights, NSW, Australia; ^7^ Human Physiology and Sports Physiotherapy Research Group, Vrije Universiteit Brussel, Brussels, Belgium; ^8^ Department of Quality of Life Sciences, University of Bologna, Rimini, Italy

**Keywords:** cognition, neuroscience, brain, psychology, physiology

**Editorial on the Research Topic** Mental fatigue and sport: from the lab to the field

Prolonged periods of cognitive activity or high cognitive demand for a short time can induce mental fatigue (1). This psychobiological state is characterized by an increased sensation of tiredness and low energy, impaired executive functions (e.g., sustained attention, inhibitory control, and working memory), and altered neural brain activity (2). More than 100 years ago, in 1891, the Italian scientist Angelo Mosso reported the first evidence that mental fatigue could impair human physical performance (3). He showed that performing lectures and oral exams for the whole morning reduced the muscular endurance of two of his colleagues in a repetitive middle finger flexion task. Much later, in 2009, Marcora et al. published a seminal study showing that the mental fatigue experimentally induced by 90 min of effortful cognitive activity reduced the time to exhaustion in a constant-load cycling test (4). Since then, several research groups have investigated the effects of mental fatigue on many sports performance-related capabilities, such as endurance performance, maximal strength, power and speed production, technical-tactical and perceptual-cognitive skills (2, 5, 6). The present editorial article summarizes the main findings of the studies submitted to our Research Topic, extending our knowledge in this developing field.

Chen et al. demonstrated the recent increased interest in studies about mental fatigue and athletic performance. These researchers conducted a bibliometric analysis of studies on such a theme in the Web of Science database core collection published from 2001 to 2021. They mapped the main disciplines that studied such an issue, the countries and institutions that conducted more research in the field, and the scientific journals and authors that were most cited. The authors found that publications have significantly increased in the last five years. By keywords co-occurrence clustering, they could also describe the hot research topics in the field, which include the application of EEG technology to obtain an objective indicator of mental fatigue, the search for pharmacological or non-pharmacological interventions against mental fatigue, and the comprehension of the mental fatigue effect on endurance performance, technical skills, and sports-related decision-making.

Systematic reviews and meta-analyses have unanimously demonstrated a deleterious effect of mental fatigue on endurance performance (1, 2, 6–8). This deleterious effect does not seem influenced by individual features, such as sex, age, or physical fitness (6, 9). Whilst the negative effect of mental fatigue on endurance performance is quite clear, mental fatigue does not seem to affect neuromuscular performance during short-duration and repeated maximal efforts (2, 5, 10). Recently, some studies have shown a negative mental fatigue effect on the performance of motor and perceptual-cognitive skills in different sports (11–13). Accordingly, Yuan et al. did a systematic scoping review to investigate the mental fatigue effect on sport-specific motor performance among team-sports athletes. The 12 retrieved studies examined team sports athletes from soccer, basketball, cricket, rugby, field hockey, volleyball, and Australian football. The authors found a deleterious effect of mental fatigue on intermittent endurance exercise capacity and the following skill variables: ball loss, passing and shooting errors, interceptions, and successful tackles. In addition, Cao et al. systematically reviewed the scientific literature on the effect of mental fatigue on basketball players' physical, technical, tactical, and cognitive performance. They found that mental fatigue impaired free throws, three-point shots, and total turnovers. Interestingly, the researchers did not find studies investigating mental fatigue's influence on basketball players' physical and tactical performance.

Many sport-related (e.g., travel, pre-match activity, and team talks) and non-sport related (e.g., education, excessive use of social media or video games) situations involve a high cognitive demand that can potentially generate a mental fatigue state (14). The sport itself poses high cognitive demands on the athletes and thus can cause mental fatigue (15). Therefore, it is crucial to develop, investigate and implement strategies to counteract the adverse effects of mental fatigue (16). For example, Sun et al. investigated in mentally-fatigued university soccer players whether exposure to a sequence of natural setting photographs could improve a video-based decision-making task performance involving real-soccer match situations. They found that the group that viewed 12.5 min of natural scenes improved the accuracy and reaction time in the soccer decision-making task as compared to the baseline values obtained before the mental fatigue induction. Such an effect was not observed in the control groups exposed to urban pictures. Moreover, the researchers found that the exposure effect to natural scenes seems to be time-dependent since the groups exposed for 4.17 and 8.33 min to the pictures did not change the soccer decision-making task performance from baseline values.

The underlying mechanisms for the harmful effects of mental fatigue on subsequent endurance performance, technical skills, and sports-related decision-making still need to be elucidated (17–19). Mangin et al. put some light on this topic. They submitted 83 subjects to a 30-min modified Stroop or a control task (watching a documentary) followed by a time-to-exhaustion handgrip exercise. Compared to the control task, the prolonged cognitive task impaired the subsequent physical performance. Interestingly, the authors found a statistically significant small correlation between the cognitive performance decrement during the 30-min modified Stroop task (time-on-task effect) and physical performance decrement in the time-to-exhaustion handgrip exercise (sequential task effect). For the authors, this relationship between the time-on-task and sequential task effects suggests that the two fatigue-related phenomena share a common mechanism but are not entirely equivalent.

To conclude, the studies presented in this Research Topic demonstrate that the research on mental fatigue continues to develop from well-controlled laboratory-based studies with high internal validity to studies in sporting settings with more real-world validity. However, sports scientists still have several important issues to address, such as the practicalities of monitoring mental fatigue in applied sporting environments and the psychobiological factors that moderate and mediate mental fatigue's deleterious effects on physical and technical-tactical performance across many different sports. These issues make such an area rich for future investigations.
